# The impact of multimorbidity on foot health outcomes in podiatry patients with musculoskeletal foot pain: a prospective observational study

**DOI:** 10.1186/s13047-019-0346-x

**Published:** 2019-07-03

**Authors:** Gordon J. Hendry, Linda Fenocchi, Helen Mason, Martijn Steultjens

**Affiliations:** 1 Centre for Living, School of Health and Life Sciences, Glasgow Caledonian University, Cowcaddens Road, Glasgow, G4 0BA Scotland UK; 2 Yunus Centre for Social Business and Health, Glasgow Caledonian University, Cowcaddens Road, Glasgow, G4 0BA Scotland UK

**Keywords:** Multimorbidity, Comorbidity, Musculoskeletal, Foot pain, Podiatry

## Abstract

**Background:**

Multimorbidity is prevalent and adversely affects health outcomes. Foot pain is common and one of the primary reasons for utilisation of podiatry services. At present, little is known about the impact of multimorbidity on foot health and related outcomes following podiatric intervention. The aims of this study were to evaluate whether there is a difference in foot health outcomes following exposure to podiatric foot care for people with and without multimorbidity; and ii) to evaluate whether the presence or absence of multimorbidity affects patients’ perceptions of change in foot pain.

**Methods:**

The PROMFoot study is a prospective cohort study of adults with a new episode of foot pain attending the podiatry service within the NHS Greater Glasgow and Clyde health board. Baseline medical comorbidity status (no condition, single condition, multiple conditions), longitudinal data on foot health measured using the Foot Health Status Questionnaire (FHSQ), and patient rating of change scores for foot pain were obtained from the PROMFoot study at baseline, and 3 and 6 months after podiatric intervention. Foot health scores (pain, function, footwear and general foot health) and perceptions of change for foot pain were compared between comorbidity groups.

**Results:**

A total of 115 participants (59% female) with a mean age of 55 years were included. Multimorbidity was common, affecting 61 participants (53%); while 28 (24.3%) and 26 (22.6%) reported single or no medical comorbidities respectively. Significantly worse foot health scores for all FHSQ domains were observed for the multimorbidity group at baseline, 3 and 6 months. Change scores for foot pain were similar between groups and demonstrate modest improvements, however multimorbidity group membership was strongly associated with a perceptions of change in foot pain. Multimorbidity was independently associated with poorer foot function outcomes at 3 months, and poorer foot pain and foot function outcomes at 6 months.

**Conclusions:**

Multimorbidity was associated with poor foot health outcomes and lower rates of self-perceived improvement in foot pain over 6 months following podiatric intervention in a sample of patients attending podiatric biomechanics clinics for a new episode of foot pain.

**Electronic supplementary material:**

The online version of this article (10.1186/s13047-019-0346-x) contains supplementary material, which is available to authorized users.

## Background

Foot pain is reported as common in the general population with prevalence estimates ranging from 17 to 30% [1]. Recent research suggests that multimorbidity may be an important correlate of foot pain [2], and the presence of multiple chronic diseases has been identified as a strong predictor of podiatry service utilisation [3]. Multimorbidity, defined as the co-existence of two or more medical conditions is a major national and international health concern [4]. It affects 25% of the Scottish population [4] and approximately 50 million people in the European Union, and its prevalence is rising [5, 6]. Multimorbidity is strongly associated with increasing age, with a Scottish prevalence of 65% in those aged 65–84, increasing to 82% in those aged 85 or over [4]. It is also strongly associated with social deprivation, and occurs on average around 10–15 years earlier in the most deprived compared to the least deprived areas [4]. People with multimorbidity tend to have a lower quality of life and poorer health outcomes than people with a single condition [7]. It is a major cause of work disability and is a significant burden on the health service due to patients’ complex and long-term care needs [6, 8]. Patients with multimorbidity are also particularly vulnerable to treatment burden, where poor health and disease symptoms impact on their ability to attend healthcare appointments, adhere to medical and non-medical management regimes, and undertake physical activity [6].

Patterns of diseases in multimorbidity can be highly variable as it can occur due to simple co-occurrence by chance, exposure to shared risk factors, and/or a pathogenic link between conditions [6]. For example, risk factors for musculoskeletal (MSK) disorders such as obesity are often shared with other prevalent chronic long-term conditions such as type 2 diabetes [6, 9]. Further, pathogenic links have been identified between MSK disorders such as inflammatory joint diseases and cardiovascular disease [6, 10]. Along with cardiovascular diseases and mental health problems, MSK disorders represent a major multimorbidity cluster [11]. For these reasons, MSK disorders are a major feature of multimorbidity.

At present, little is known about the impact of multimorbidity on foot health and related outcomes following podiatric intervention. For MSK foot pain, the current literature is dominated by studies which focus on single diseases (such as rheumatoid arthritis) or non-disease-specific risk factors (such as age, gender and/or obesity) and their associations with foot health outcomes such as foot pain and/or function [12, 13]. With regards to podiatry, the majority of podiatric foot care, research and education largely conforms to a single-disease framework which may be suboptimal given the rapidly increasing prevalence of multimorbidity. At present, it is unclear how multimorbidity impacts on foot health and outcomes following exposure to podiatric interventions. Accordingly, the aims of this exploratory study were to i) evaluate whether there is a difference in foot health outcomes following exposure to podiatric foot care for people with and without multimorbidity; and ii) to evaluate whether the presence or absence of multimorbidity affects patients’ perceptions of change in foot pain.

## Methods

### Participants and setting

Participants in this study are from the Patient Reported Outcome Measures Foot (PROMfoot) Study. In short, PROMfoot is a longitudinal observational cohort study that was designed to evaluate measurement properties of four different foot-specific and generic patient reported outcome measures (PROMs) including the Foot Health Status Questionnaire (FHSQ), the Foot Function Index, the EuroQoL 5-dimensional questionnaire (EQ5D-5 L), and the Short-form 6-dimension questionnaire. The PROMfoot study was conducted within the National Health Service (NHS) Greater Glasgow and Clyde (GG&C) Health Board region. Patients with a new episode of foot pain who were referred to NHS GG&C Podiatric Biomechanics Clinics were the target population. Referrals to NHS GG&C Podiatric Biomechanics Clinics are largely comprised of patients with complex musculoskeletal foot pathology. Patients are allocated at the discretion of the podiatrist responsible following vetting of written referrals from general practice, orthopaedics, other health professions, or internal escalation following initial podiatry treatment. Patients were eligible to participate if they i) were scheduled to attend as a new patient for treatment at a podiatric biomechanics clinic within NHS GG&C; ii) self-reported current foot pain; iii) were aged 18 years or more; and iv) were willing and able to provide written informed consent. Patients were not eligible to participate if they failed to meet any of the above inclusion criteria. Ethical approval was obtained from the South East Scotland NHS Research Ethics Committee (16/SS/0193) on 2nd November 2016. Recruitment was undertaken between January and December 2017. For the purposes of addressing the aims of this study, longitudinal FHSQ data from PROMfoot are reported.

### Recruitment

Potentially eligible participants were identified by screening NHS GG&C Podiatric Biomechanics clinic lists using the TrakCare electronic patient management system by an NHS GG&C Podiatrist. Potentially eligible participants were sent an invitation letter and a study participant information sheet, and willing participants were invited to contact the researcher (LF). Telephone-based screening was undertaken to confirm study eligibility and patients’ willingness to participate. Patients who were willing to take part were invited to return signed consent forms to the researcher to confirm their participation in the study and complete baseline assessments via completion of a postal or web-based survey, according to their personal preference. Enrolment was confirmed upon receipt of written consent and completed baseline data.

### Data collection baseline

Study data were collected and managed primarily using the Research Electronic Data Capture (REDCap) secure electronic data capture tool hosted at Glasgow Caledonian University [14]. Study data obtained using paper based forms and returned via mail were manually entered into the REDCap database by the researcher.

Baseline demographic data collected included age (years), sex, employment status (full-time, part-time, voluntary, looking for work, student, looking after home/family, wholly retired, permanently unable to work, other, prefer not to answer), and self-reported height and weight for calculation of body mass index (BMI). Participants’ post codes were collected in order to calculate indices of social deprivation [15–17]. These were expressed as lowest 2 quintiles (most deprived) versus upper 3 quintiles (least deprived). Self-reported health-related quality of life (HRQoL) was measured as a baseline descriptor of health status and evaluated using the Euro quality of life (Euroqol) five dimension 5 level questionnaire (EQ-5D-5 L) and 100 mm visual analogue scale (EQ-VAS) (higher scores indicating better health) [18]. Medical conditions/comorbidities were evaluated using the Self-Administered Comorbidity Questionnaire (SCQ), a valid and reliable questionnaire which requires no prior medical knowledge for the self-report of comorbidity [19]. The SCQ scores the presence or absence of comorbid conditions, whether or not the participant receives treatment for the condition(s), and whether the condition(s) limits activities [19]. For the purpose of this study, multimorbidity was defined as the coexistence of two or more chronic conditions [20]. In order to address the impact of multimorbidity on primary and secondary outcome variables, the presence or absence of single medical conditions or multimorbidity was calculated using baseline SCQ responses to create 3 groups; 1) no conditions (*n* = 0 conditions), 2) single condition (*n* = 1 condition), and 3) multimorbidity (> 1 condition). Foot pain severity experienced during the previous week was measured as a baseline descriptor using a numerical rating scale (NRS) from 0 to 10 (0 no pain, 10 worst pain possible). Regional foot pain location (hindfoot, forefoot, toes, ball, arch, heel, nails) was evaluated using a foot pain map developed previously and widely used for epidemiological research [21].

### Primary and secondary outcomes

The primary outcome of interest was foot health at 3 and 6 month follow-ups, which was evaluated using the Foot Health Status Questionnaire (FHSQ), a valid and reliable 13-item questionnaire with four domains including pain, function, footwear, and general foot health [22, 23]. The FHSQ is completed using a 5-point Likert scale, and scored by transforming to a scale ranging from 0 (poorest foot health) to 100 (best foot health) [22, 24]. The secondary outcome of interest was participants’ perceptions of change in foot pain, measured using a 7-point patient rating of change scale (PRCS) for foot pain (very much worse to very much improved) [25] from baseline to 3 and 6 month follow-ups respectively.

### Follow-up data collection

Follow-up study data were captured at 3 and 6 months after baseline measurements. Three and 6-month data included the 4 domains of the FHSQ and the 7-point PRCS. Podiatric foot care treatments received and self-care undertaken over the 6-month study period were recorded for each participant using a standard check-box list (foot orthoses, footwear advice/modifications, medications, rest, exercises, physical therapies, and acupuncture) and free text box for other treatments.

### Statistical analyses

Demographic and general health characteristics were summarized using descriptive statistics. Continuous data were screened for normality of distributions using the Kolmogorov-Smirnov test. Continuous variables were summarised using means and standard deviations (SD), and medians and inter-quartile ranges (IQR) where appropriate. For categorical and ordinal data, proportions were calculated and expressed as absolute frequencies (n) and percentages (%). Ordinal data from the PRCS were dichotomised and recoded as a categorical variable (improvement versus no change/deterioration). Differences in demographic and general health characteristics between groups were evaluated using Kruskal-Wallis and Mann-Whitney post hoc tests where appropriate.

Differences between groups for discrete FHSQ domain scores at baseline, 3 and 6 months, and change scores between baseline, 3 and 6 months were evaluated using Kruskal-Wallis tests and Mann-Whitney post hoc tests where appropriate. To identify associations between multimorbidity status and participants’ self-perception of change in foot pain (dichotomised PRCS), Pearson’s chi-square tests were performed and presented as 3 × 2 contingency tables. Chi square test effect sizes were calculated using Cramer’s V. Multivariate linear regression analyses were undertaken to identify associations between multimorbidity status and FHSQ domain scores at 3 and 6 months with and without adjustment for covariates. All tests were two-tailed and *p* values < 0.05 were considered to indicate statistical significance. All analyses were undertaken using IBM® SPSS® version 25.

Given the prospective nature of the study, some loss to follow-up was anticipated. Missing data were accounted for using 3 approaches. Secondary missing data analyses included 1) comparison of baseline demographic characteristics between the complete (referred to as closed cohort) and incomplete cohorts (open cohort); 2) graphical explorations of primary outcome data at discrete time points and change scores over time for closed and open cohorts; and 3) sensitivity analyses with repetition of primary and secondary outcome data analyses for the closed cohort (see Additional files 1, 2, 3, 4, 5, 6 and 7). The primary method of analysis was open cohort with no imputation of missing data, supplemented with secondary missing data analyses provided above to aid interpretation.

## Results

### Study population

**Fig. 1:**
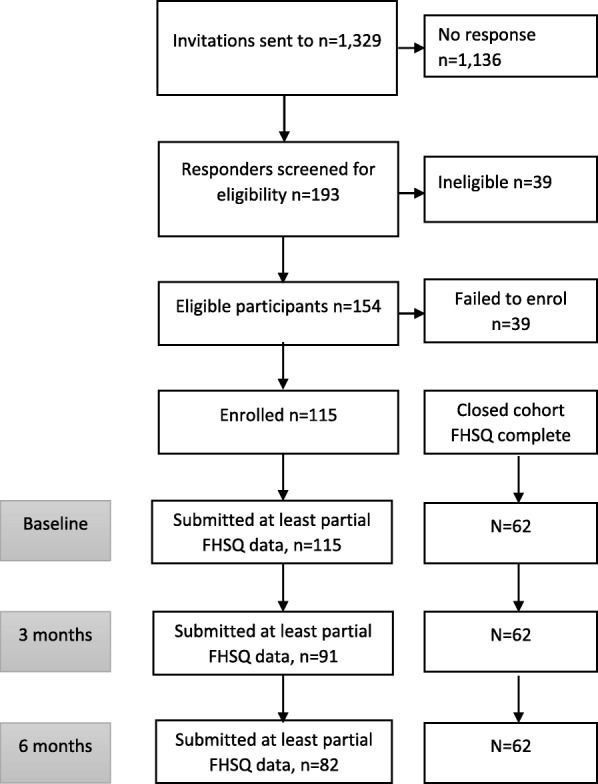
Study participant flow chart

Of the 1329 invitations sent, 193 individuals responded (14.5%). Of these, 154 (79.8%) were eligible, and of these 115 (74.7%) were enrolled (Fig. 1). Of the 115 participants, 59% were female and the mean (SD) age was 55 (11.6) years. At least partial FHSQ data (defined as completion of at least 1 FHSQ domain) was available for 115 participants at baseline, reducing to 91 (79%) at 3 months, and 82 (71%) at 6-month follow-up. A total of 62 participants (54%) had complete baseline, 3 and 6 months’ follow-up data for all FHSQ domains.

Participant demographic characteristics and baseline clinical characteristics are summarised in Tables 1, 2 and 3. The sample was typically overweight or obese, with a median (IQE) BMI of 29.9 (19.2); in a state of suboptimal HRQoL; and approximately 42% were classified in the lower 2 quintiles (most deprived) of social deprivation. Approximately half of participants were in paid full-time or part time employment (48.7%), whilst over one-third (34.8%) were either wholly retired or permanently unable to work.

**Table 1:** Demographics

	Whole sample	No condition	Single condition	> 1 Condition
Number of participants, n (%)	115 (100)	26 (22.6)	28 (24.3)	61 (53)
Age in years, mean (SD)	55 (11.56)	53.4 (9.5)	52.4 (12.8)	56.95 (11.6)
BMI, median (IQR)	29.9 (19.2)	28.8 (19.7)	27.7 (21.6)	32.9 (17.8)†
Sex ratio (female:male)	68:45	17:9	14:13	37:23
EQ-5D-5 L Index, mean (SD)	0.55 (0.31)	0.73 (0.18)	0.69 (0.17)	0.42 (0.34) ‡‡
EQ-5D-5 L VAS, mean (SD)	66.69 (25.74)	78.62 (18.72)	75.3 (22.05)	57.8 (26.75) ‡‡
SIMD lowest 2 quintiles, n (%)	48 (41.7)	12 (46.2)	7 (25)	29 (47.5)
Employment status, n (%)				
Paid or self-employed full time	39 (33.9)	11 (42.3)	14 (50)	14 (23.0)
Paid or self-employed part time	17 (14.8)	4 (15.4)	4 (14.3)	9 (14.8)
Voluntary work	1 (0.9)	1 (3.8)	0	1 (1.6)
Looking for work	2 (1.7)	7 (26.9)	0	2 (3.3)
Student in further or higher education	2 (1.7)	1 (3.8)	2 (7.1)	0
Looking after the home or family	4 (3.5)	1 (3.8)	1 (3.6)	2 (3.3)
Wholly retired	30 (26.1)	7 (26.9)	6 (21.4)	17 (27.9)
Permanently unable to work	10 (8.7)	1 (3.8)	0	9 (14.8)
Other	5 (4.3)	1 (3.8)	0	4 (6.6)
Prefer not to answer	2 (1.7)	0	0	2 (3.3)

Mann-Whitney tests significant at ‡‡*p* < 0.01 for Single Condition versus > 1 conditions group comparison

Mann-Whitney tests significant at †*p* < 0.05 for No conditions versus > 1 conditions comparison

*SD* Standard deviation, *IQR* Interquartile range, *BMI* Body mass index, *SIMD* Scottish Index of Multiple Deprivation, *n* number of participants, *EQ-5D-5 L* EuroQoL 5 Dimensions 5 level questionnaire, *VAS* Visual analogue scale

**Table 2:** Comorbidities

Condition, n (%)	Diagnosed with condition	Receiving treatment for condition	Condition limits activities
Heart disease	12 (10.4)	10 (8.7)	6 (5.2)
High blood pressure	23 (20)	18 (15.7)	6 (5.2)
Lung disease	11 (9.6)	8 (7.0)	4 (3.5)
Diabetes	7 (6.1)	4 (3.5)	3 (2.6)
Ulcer/stomach disease	13 (11.3)	11 (9.6)	6 (5.2)
Kidney disease	3 (2.6)	1 (0.9)	2 (1.7)
Liver disease	3 (2.6)	1 (0.9)	1 (0.9)
Anaemia or other blood disease	10 (8.7)	6 (5.2)	6 (5.2)
Cancer	6 (5.2)	2 (1.7)	2 (1.7)
Depression	20 (17.4)	11 (9.6)	15 (13.0)
Osteoarthritis or degenerative arthritis	38 (33.0)	24 (20.9)	32 (27.8)
Back Pain	50 (43.5)	22 (19.1)	34 (29.6)
Rheumatoid or inflammatory arthritis	9 (7.8)	5 (4.3)	6 (5.2)
Other medical problems	44 (38.3)	–	–
Rheumatic or musculoskeletal diseases^a^	7 (6.1)	–	–
Neurological conditions^b^	9 (7.9)	–	–
Hypo/hyper thyroidism	5 (4.4)	–	–
Asthma	4 (3.5)	–	–
Sleep apnoea	3 (2.6)	–	–
Other	16 (13.9)	–	–

^a^gout, mixed connective tissue disease, Sjogren’s syndrome, ankylosing spondylitis, fibromyalgia, osteopenia, and benign hypermobility (all *n* = 1)

^b^stroke/transient ischaemic attack (*n* = 3), trigeminal neuralgia, multiple sclerosis, Parkinson’s disease, epilepsy, polio, and neuropathy (all *n* = 1)

*N* Number of participants

**Table 3:** Baseline foot pain characteristics

	Whole sample (*n* = 115)	No condition (*n* = 26)	Single condition (*n* = 28)	> 1 condition (*n* = 61)
Foot pain NRS 0–10, median (IQR)	6 (4)	5 (2.25)	6 (2.75)	8 (4)
Foot pain region, n (%)				
Hindfoot	50 (43.5)	8 (30.8)	5 (17.9)	37 (60.7)
Forefoot	61 (53.0)	15 (57.7)	11 (39.3)	35 (57.4)
Toes	59 (51.3)	9 (34.6)	14 (50.0)	36 (59.0)
Ball	56 (51.3)	12 (46.2)	14 (50)	30 (49.2)
Arch	55 (47.8)	9 (34.6)	9 (32.1)	37 (60.7)
Heel	49 (42.6)	13 (50.0)	6 (21.4)	30 (49.2)
Nails	12 (10.4)	1 (3.8)	1 (3.6)	10 (16.4)

*NRS* Numerical rating scale, *IQR* Interquartile range, *n* number of participants

Twenty-six participants (22.6%) reported having no medical condition, while 28 (24.3%) and 61 (53%) reported having a single or multiple conditions respectively (Table 2). The median (range) for total number of conditions per participant was 2 (0–11). Back pain was the most common comorbid medical condition reported by participants (43.5%), followed by osteoarthritis (33.0%), high blood pressure (20.0%), depression (17.4), ulcer/stomach disease (11.3%) and heart disease (10.4%). Of the comorbid medical conditions with a sample point prevalence at baseline greater than 10%, osteoarthritis was the condition which proportionally resulted in limitation of activities for most participants (32/38, 84.2%), followed by depression (15/20, 75.0%), back pain (34/50, 68.0%), heart disease (6/12, 50%), ulcer/stomach disease (6/13, 46.2%) and high blood pressure (6/23, 26.1%). Moderate proportions of participants with osteoarthritis (36.8%), back pain (66%) and/or depression (45%) were not receiving treatment for their condition.

At study baseline, all participants presented with foot pain and this ranged generally from moderate to severe, with a median (IQR) of 6/10 (4) (Table 3). Foot pain was common, with between 42.6–53% of participants reporting pain affecting the heel, hindfoot, arch, ball, toes, and forefoot regions. Pain at the nails was less frequently reported (10.4%). Median (IQR) values for foot health at baseline suggest that all FHSQ domains were significantly impaired including FHSQ pain (35, 16.86–60.0), function (62.5, 25.0–81.25), footwear (33.33, 16.67–58.33) and general foot health (25.0 0–60.0).

### Morbidity group demographic and clinical characteristics

Median age and BMI were higher in the group with multiple medical conditions compared to no conditions (age *p* = 0.099 not significant (NS), BMI *p* = 0.041) and single condition group (age *p* = 0.111 NS, BMI *p* = 0.084, NS) (Table 1). Participants in the multiple conditions group reported lower EQ-5D-5 L Index and VAS scores than no conditions (*p* < 0.001, *p* = 0.001) and single conditions groups (*p* < 0.001, *p* = 0.004) (Table 1). Absolute and relative frequencies suggest lower rates of paid full-time employment, higher rates of social deprivation, and being permanently unable to work in the multimorbidity group (Table 1). Participants with multiple conditions typically reported more severe foot pain, and proportionally more foot pain affecting the hindfoot, toes, arch, and/or nails regions of the foot (Table 3).

### Closed versus open demographic and clinical characteristics

Baseline comparisons of demographic and clinical characteristics between the closed versus open cohort suggest cohorts were similar in terms of age, sex, BMI, HRQoL and employment status (Additional file 1). The closed cohort participants reported lower rates of social deprivation.

### Treatments received between baseline and follow-up

Treatments received over the 6-month study period are summarised in Table 4. A median (IQR) of 2 (2–3) treatments were received per patient and these most frequently involved foot orthoses (47.8%), exercises (40.8%), footwear advice/modifications (34.8%), medications (27.8%), rest (26.1%), physical therapies (20.0%) and/or self-management (19.1%).

**Table 4:** Podiatry treatments received over previous 6 months

Podiatry treatments received over previous 6 months	Number of participants n (%)
Foot orthoses	55 (47.83)
Footwear advice/modifications	40 (34.78)
Medications	32 (27.83)
Rest	30 (26.09)
Exercises	47 (40.87)
Physical therapies	23 (20)
Acupuncture	3 (2.61)
Other^a^	30 (26.09)
Self-management	22 (19.13)
Number of treatments received per patient (median, IQR)	2 (2–3)

^a^Referral (*n* = 5), steroid injection (*n* = 3), rocker boot, nail care, trans-electrical nerve stimulation, dome pad

*N* Number of participants, *IQR* Interquartile range

### Foot health differences between comorbidity groups

**Fig. 2:**
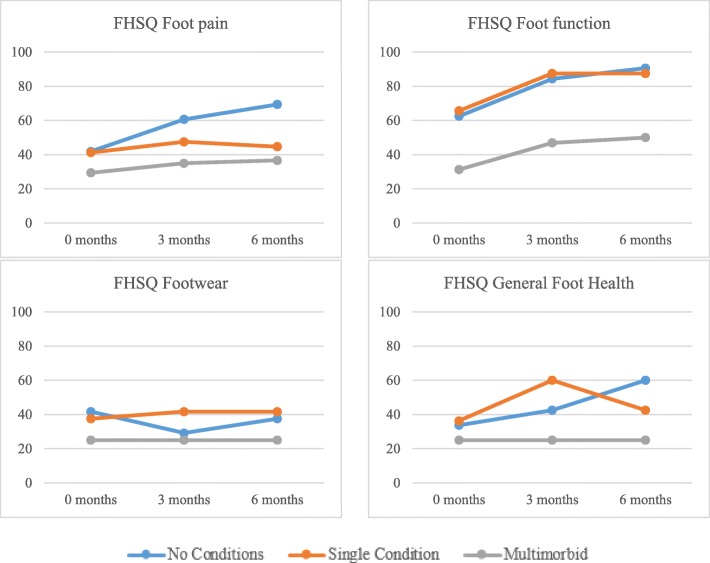
Median fhsq scores for comorbidity groups at 0, 3 and 6 months

Between group comparisons for foot health at discrete time points (baseline, 3 and 6 month follow-ups) are presented in Table 5 and Fig. 2. The multimorbidity group exhibited worse foot health scores for each domain at each discrete time point. The single conditions group had worse FHSQ foot pain and FHSQ foot health outcomes at 6 months, but was similar to the no conditions group for FHSQ footwear and FHSQ foot function. Statistically significant differences were observed between groups for FHSQ foot function at baseline, and 3 and 6 month follow-ups, with the multimorbidity group having significantly worse foot function than the no conditions and single conditions groups (all *p* < 0.01). At 6 months, FHSQ pain scores were significantly worse in the multimorbidity versus no conditions group (*p* < 0.01), and FHSQ footwear scores were significantly worse in the multimorbidity versus single conditions group (*p* < 0.01). Statistically significant differences were observed between groups for FHSQ general foot health at baseline, 3 months and 6 months, with the multimorbidity group having significantly worse scores than the no conditions (*p* < 0.01) and single conditions group (*p* < 0.05) at baseline, and the no conditions group at 3 and 6 months (both *p* < 0.05).

**Table 5:** Group comparisons for FHSQ domains at baseline, 3 and 6 month follow-ups

	FHSQpain, median (IQR)	FHSQfunction, median (IQR)	FHSQfootwear, median (IQR)	FHSQhealth, median (IQR)
Baseline				
No condition	41.88 (43.13)	62.5 (40.63)**	41.67 (39.58)	33.75 (38.13)**
Single condition	41.25 (41.25)	65.63 (31.25)**‡‡	37.5 (41.67)	36.25 (44.38)**‡
> 1 conditions	29.38 (48.75)	31.25 (53.13)**††	25.0 (39.58)	25.0 (42.5)**††
3 months				
No condition	60.63 (39.69)	84.38 (28.13)**	29.17 (31.25)	42.5 (53.75)*
Single condition	47.5 (46.25)	87.5 (31.25)**‡‡	41.67 (58.33)	60.0 (35.0)*
> 1 conditions	35.0 (43.13)	46.88 (64.06)**††	25.0 (54.17)	25.0 (60.0)*†
6 months				
No condition	69.38 (26.09)*	90.63 (21.88)**	37.5 (52.08)*	60.0 (66.25)*
Single condition	44.69 (48.91)*	87.5 (31.25)**‡‡	41.67 (50.0)* ‡‡	42.5 (60.0)*
> 1 conditions	35.63 (48.91)*††	50.0 (59.38)**††	25.0 (45.83)*	25.0 (51.25)*†

Kruskall-Wallis tests significant at **p < 0.01 and *p < 0.05

Post-hoc Mann-Whitney tests significant at ‡‡*p* < 0.01 and ‡*p* < 0.05 for Single Condition versus > 1 conditions group comparison

Post-hoc Mann-Whitney tests significant at ††*p* < 0.01 and †*p* < 0.05 for No conditions versus > 1 conditions comparison

*FHSQ* Foot health status questionnaire, *IQR* Interquartile range

Between group changes scores are presented in Table 6. There were no significant differences between groups for change scores for any FHSQ domains between 0 and 3 or 0–6 months (all *p* > 0.05). Modest improvements in FHSQ pain were observed for each group between 0 and 3 and 0–6 months. Modest improvements in FHSQ function were observed for no conditions and single condition groups only. Median scores of 0 suggest that FHSQ general foot health did not improve for any group. The FHSQ footwear domain scores improved between 0 and 6 months for the single condition group only.

**Table 6:** Group comparisons for change scores for FHSQ domains between baseline and 3 months and baseline and 6 month follow-ups

	FHSQ pain, median (IQR)	FHSQ function, median (IQR)	FHSQ footwear, median (IQR)	FHSQ health, median (IQR)
0 to 3 months				
No condition	−6.26 (35.94)	− 6.23 (21.88)	0 (22.91)	0 (9.38)
Single condition	−5.63 (16.88)	−6.23 (21.88)	0 (25.0)	0 (21.25)
>1 conditions	−6.26 (13.76)	0 (18.75)	0 (33.33)	0 (12.5)
0 to 6 months				
No condition	−11.88 (45.0)	−18.75 (40.63)	0 (43.75)	0 (31.25)
Single condition	−1.57 (41.10)	−9.38 (25.0)	−8.33 (25.0)	0 (37.5)
>1 conditions	−6.26 (22.81)	0 (12.5)	0 (25.0)	0 (18.75)

No significant differences between groups for changes scores, Kruskall-Wallis tests

*FHSQ* Foot health status questionnaire, *IQR* Interquartile range

### Multivariate associations between morbidity group and FHSQ outcomes

In unadjusted analyses, multimorbidity was significantly associated with lower (poorer) scores for FHSQ pain (*p* < 0.01), function (*p* < 0.01) and general health (*p* < 0.04) at 3 and 6 month follow ups (Table 7). In analyses with adjustment for baseline corresponding FHSQ domain score, age, BMI and sex, multimorbidity was significantly independently associated with lower (poorer) scores for FHSQ function (*p* = 0.01) at 3 months, and FHSQ pain (*p* = 0.02) and FHSQ function (*p* < 0.01) at 6 months (Table 7).

**Table 7:** Associations between multimorbidity and FHSQ pain and function domains

Variable	FHSQ pain 3 months			FHSQ pain 6 months		
Unadjusted	*B* (95% confidence interval)	*SE B*	*β*	*B*	*SE B*	*β*
Multimorbidity	−17.95 (−32.92, − 2.99)	7.53	− 0.32*	−25.95 (− 41.68, − 10.23)	7.90	−0.48**
Single condition	− 11.13 (− 28.66, 6.40)	8.82	− 0.17	− 18.20 (− 35.72,-0.68)	8.80	− 0.30*
Adjusted						
Multimorbidity	−2.64 (− 13.98, 8.71)	5.70	− 0.05	− 17.17 (− 31.02, − 3.31)	6.94	− 0.32*
Single condition	−3.35 (− 16.14, 9.45)	6.42	− 0.05	− 13.61 (− 28.72, 1.16)	7.40	−0.23
Baseline FHSQ pain	0.85 (0.67, 1.04)	0.09	0.77**	0.63 (0.44, 0.83)	0.10	0.60**
Age	− 0.35 (− 0.76, 0.06)	0.21	−0.14	− 0.14 (− 0.57, 0.30)	0.22	−0.06
BMI	0.13 (−0.23, 0.49)	0.18	0.06	−0.01 (− 0.43, 0.41)	0.21	− 0.01
Sex	− 0.11 (− 9.5, 9.30)	4.72	− 0.00	−1.88 (− 12.57, 8.82)	5.40	− 0.03
	FHSQ function 3 months			FHSQ function 6 months		
Unadjusted	*B* (95% confidence interval)	*SE B*	*β*	*B*	*SE B*	*β*
Multimorbidity	−33.63	8.11	−0.51**	−35.36	8.67	−0.55**
Single condition	−5.36	9.48	−0.07	− 5.07	9.68	−0.07
Adjusted						
Multimorbidity	−16.70 (−29.72, −3.67)	6.54	−0.25*	−21.71 (−34.02, −9.39)	6.17	− 0.34**
Single condition	−8.60 (− 22.34, 5.15)	6.90	− 0.11	−10.84 (−23.93, 2.26)	6.56	− 0.16
Baseline FHSQ function	0.75 (− 0.28, 0.58)	0.08	0.71**	0.73 (0.58, 0.88)	0.07	0.71**
Age	0.15 (−0.28, 0.58)	0.22	0.05	−0.16 (− 0.55, 0.22)	0.19	− 0.06
BMI	0.03 (−0.35, 0.41)	0.19	0.01	−0.07 (− 0.43, 0.28)	0.18	− 0.03
Sex	3.98 (−5.86, 13.81)	4.94	0.06	3.98 (−5.29, 13.22)	4.64	0.86

FHSQ Pain 3 months*, R*^2^ = 0.06 for unadjusted, *R*^2^ = 0.56 for adjusted; FHSQ Pain 6 months, *R*^2^ = 0.12 for unadjusted, *R*^2^ = 0.46 for adjusted

FHSQ Function 3 months*, R*^2^ = 0.22 for unadjusted, *R*^2^ = 0.65 for adjusted; FHSQ Function 6 months, *R*^2^ = 0.25 for unadjusted, *R*^2^ = 0.69 for adjusted

Significant at **p < 0.01 and *p < 0.05

*FHSQ* Foot health status questionnaire, *B* Beta coefficient, *SE β* Coefficient standard error, *β* Standardised beta coefficient

### Perception of change in foot pain group comparisons

Group comparisons for PRCS scores between 0 and 3 and 0–6 months are presented in Table 8 Proportionally, more participants in the multimorbidity group reported their foot pain had either deteriorated or had not changed between 0 and 3 and 0–6 months. Proportionally, more participants in the no conditions group reported that their foot pain had improved between 0 and 3 and 0–6 months. Group membership was significantly associated with the perception of change in foot pain between 0 and 3 (X^2^ (2), 6.61, *p* = 0.037, medium effect size) and 0–6 months (X^2^ (2), 6.90, *p* = 0.032, medium-to-large effect size).

**Table 8:** Contingency tables for associations between multimorbidity group membership and PRCS improvement versus no change/deterioration

	No change/ deteriorated	Improved	Cramer’s V	*p*-value
0 to 3 months (*n* = 94)				
No condition	7 (35.0)	13 (65.0)	0.265	0.037*
Single condition	11 (50.0)	11 (50.0)	–	–
Multimorbidity	35 (67.3)	17 (32.7)	–	–
0 to 6 months (*n* = 82)				
No condition	4 (28.6)	10 (71.4)	0.29	0.032*
Single condition	11 (47.8)	12 (52.2)	–	–
Multimorbidity	30 (66.7)	15 (33.3)	–	–

*N* Number of participants

### Sensitivity analyses

Closed versus open cohort sensitivity analyses are presented in Additional files 2, 3, 4, 5 and 6. Foot health data at discrete time points was similar between closed and open cohorts, with the multimorbidity group having lower median FHSQ domain scores at each time point. Some subtle differences were observed for the closed cohort for no conditions versus single condition groups, where the single condition group had better scores for some foot health domains including FHSQ pain, FHSQ function and FHSQ footwear at baseline, and FHSQ general foot health at 3 months. Closed cohort inferential statistical results were largely consistent with open cohort analyses with the exception of FHSQ pain at 3 months (significantly poorer in multimorbidity group versus no condition, *p* < 0.05), FHSQ pain at 6 months (no statistically significant difference), and FHSQ general foot health at baseline (no statistically significant difference). Analyses of change scores were consistent between closed and open cohorts with the exception of FHSQ footwear, which was significantly worse in the multimorbidity group versus no conditions group for the closed cohort. In spite of similarly high proportions of multimorbidity group reporting no improvement relative to no conditions and single condition groups within the closed cohort, associations between group membership and perceptions of change in foot pain were not statistically significant.

## Discussion

The results of this study demonstrate that people with multimorbidity had significantly poorer foot health for each domain of the FHSQ than people without, both before and after podiatric intervention. When adjusted for corresponding baseline domain FHSQ score, age and BMI, multimorbidity was independently associated with worse FHSQ outcomes for foot function at 3 months, and foot pain, foot function and footwear at 6 months. The multimorbidity group did not differ significantly from comparator groups for FHSQ domain change scores following intervention. Only modest improvements were observed for each group for the foot pain domain, with improvements in foot function observed in the no conditions and single conditions groups only. These improvements generally approached published minimal important difference (MID) values for the FHSQ [25]. However, relative to the comparator groups the multimorbidity group typically perceived that their foot pain did not improve following exposure to podiatric foot care. This may be explained in part by participants in the multimorbidity group having poorer foot health at baseline, and remaining in states of relatively poorer foot health at 3 and 6 month follow-ups in spite of similar magnitudes of improvement in foot pain. Alternatively, it is possible that people with multimorbidity are less able to perceive modest improvements in their foot health in the context of experiencing other unpleasant symptoms driven by their co-occurring conditions. If the latter assumption is accurate, it is likely that larger differences in FHSQ domains may be required to achieve a difference that is perceived as beneficial in this population.

There are several potential explanations for the persistent poor foot health observed in the multimorbidity group. Whilst we acknowledge that evaluation impact of specific patterns or clusters of multimorbidity on foot health were outwith the scope of the current study, we can tentatively deduce that multimorbidity may have involved the most prevalent and limiting conditions reported namely depression, osteoarthritis and back pain. These conditions have been identified previously as correlates of foot pain [2, 26, 27] and are notoriously chronic, persistent and difficult to manage effectively. However numerous combinations of medical comorbidities were observed in this population (Table 2) and further research is required to fully understand the impact of specific multimorbidity clusters and foot health. Moreover, the coexistence of mental health disorders and other functionally limiting conditions, known as mental-physical multimorbidity, has been recently recognised as a major challenge for health care providers [4]. There may be a bidirectional relationship between mental health and painful physical disorders where painful symptoms can trigger poor mental health episodes and vice-versa. In addition, physical and mental health care is generally not delivered in tandem [4], in spite of recent efforts to add behavioural components such as brief cognitive behavioural therapy and motivational interviewing to GP [28] and Allied Health Professional-delivered interventions [29, 30].

Healthcare inequalities have been identified in the primary care management of patients with multimorbidity that include insufficient appointment times, higher stress levels amongst general practitioners (GP), and lower levels of patient enablement; defined as their ability to cope with and self-manage their conditions [31]. To address these inequalities, a GP-led intervention comprised of longer appointment times and practitioner continuity; practitioner training in holistic assessments, empathy and self-management; and patient self-management support materials was evaluated in deprived areas of Glasgow, and found to be feasible and beneficial in improving quality of life [32–34]. Whilst learning tools to foster empathic, person-centred communication have been piloted in podiatry settings recently [32], standard MSK podiatric foot care largely conforms to a single-disease model which involves management of the presenting foot complaint with the possible addition of sign-posting to lifestyle management resources for health risk factors such as smoking.

The importance of self-management has been highlighted recently as a key strategy for meeting the needs of people living with multiple long-term conditions [35]. The goals of self-management are to limit the need for further disease progression and avoid the need for more care and thus healthcare utilisation [35]. The reduced ability of patients with multimorbidity to self-manage their own conditions has been highlighted as a major barrier to improved health in this population [35]. Fewer than 20% of the overall sample in the current study reported that they attempted self-management of their foot problem between baseline and 6-month follow-up. It is unclear whether or not this was driven by the negative impact of multimorbidity, or lack of promotion of self-management techniques by the podiatrists responsible for delivering foot care. Nevertheless, this suggests that more emphasis should be placed on the development and evaluation of holistic foot and general health self-management strategies in podiatry settings.

Podiatric treatments provided over the course of the study period most frequently involved provision of foot orthoses, footwear advice and exercises, which are largely consistent with a priori expectations of United Kingdom (UK) NHS MSK podiatry services. The results of this study raise questions concerning whether or not sufficient foot health benefits can be achieved in people who suffer from multimorbidity via interventions designed to address presenting foot pain complaints in isolation. The range of medical conditions observed in this study suggest that significant proportions of patients attending podiatric biomechanics services present with complex medical problems and poor general and foot health. Primary focus on foot and ankle biomechanics without consideration of other aspects of holistic management such as promotion of self-management techniques for broader health concerns may at least in part explain the poor outcomes observed. However cautious interpretation is warranted as details of treatments received were self-reported by participants as opposed to clinician-report or case note review which may have been more accurate. Nevertheless, recent primary care research has identified educational needs amongst GPs working with multimorbid patients including: - how to address low patient engagement with their health care and low health literacy [31]. As such, there may be important training needs for health professionals working in MSK services that are required to improve confidence and competence in facilitating the holistic management of MSK foot pain patients who have multimorbidity.

There are some limitations to this study that warrant further attention. Whilst the overall sample size was sufficient, the initial response rate to study invitations was lower than anticipated and as such cautious interpretation is warranted due to potential non-response bias. The implications of this are that those who responded (and those who were eligible and were subsequently enrolled) are not necessarily representative of the invited population who were eligible. It is acknowledged that not all of the 1329 patients invited would have been eligible to participate. Of those who were eligible, the recruitment rate was high at 74.7%. Whilst attrition rates were generally acceptable at less than 20% at each follow-up, some participant data sets were subject to sporadic missing data. However, loss to follow-up and missing data were accounted for collectively via 3 separate analysis techniques which suggested only modest differences between closed (full data) and open cohorts (incomplete data) and thus selection bias was concluded to be minimal. Another strength of this study was that participants were recruited from the routine NHS Podiatric Biomechanics setting by an NHS-employed podiatrist outwith the study team, and as such vulnerability to recruitment bias was minimised. Data were obtained from the NHS GG&C Health Board Region and we acknowledge that the socioeconomic composition of this region differs to that of the rest of Scotland and the UK, with higher levels of deprivation, poor health behaviours, and lower life expectancy [36–38]. This may limit our ability to generalise these findings to other health board regions in the UK. We adopted a standard definition of multimorbidity for the purposes of this study [20]. However, we acknowledge that other definitions of multimorbidity have been adopted in the literature and heterogeneous methodological criteria make comparisons difficult [11]. In addition, morbidity groups were allocated according to participant’s responses to the SCQ which is subject to some contradictory findings concerning its construct validity in certain conditions. The SCQ has been found to be accurate relative to medical notes for the majority of its items, but performs less well for self-reported comorbidities in people with rheumatic and musculoskeletal conditions such as systemic sclerosis, systemic lupus erythematosus [39], and ankylosing spondylitis [40]. However, this limitation is largely concerned with the construct validity of the overall SCQ comorbidity score which was not used in our analyses.

## Conclusions

Multimorbidity was associated with poor foot health and impacts negatively on foot health outcomes following podiatric intervention in a sample of patients attending podiatric biomechanics clinics for a new episode of foot pain. Multimorbidity was independently associated with poorer foot pain and foot function outcomes. Multimorbidity was also associated with lower rates of perceived improvement in foot pain following podiatric intervention. Current MSK foot care may be suboptimal and there are likely important training needs for podiatrists working with multimorbid patients. Further research is required to confirm associations between multimorbidity and poor foot health.

## Additional files

Additional file 1: Table of demographic characteristics of the whole sample versus the closed cohort. Table comparing demographic characteristics between the whole sample which includes some missing data, and the closed cohort with complete data at each time point. (DOCX 12 kb)

Additional file 2: Table of FHSQ domain scores for the closed cohort. Table displays FHSQ domain scores at each study time point and results of between group sensitivity analysis. (DOCX 12 kb)

Additional file 3: Table of FHSQ domain change scores scores for the closed cohort. Table displays FHSQ domain change scores at each study follow-up and results of between group sensitivity analysis. (DOCX 12 kb)

Additional file 4: Cross-tabulation contingency table of perceptions of foot pain change according to multimorbidity group. Table displays results of cross-tabulation frequencies and chi-square sensitivity analysis of associations between group membership and perceptions of foot pain change. (DOCX 12 kb)

Additional file 5: Unadjusted and adjusted associations between multimorbidity and foot health outcomes. Table displays results of multivariate linear regression analyses for associations between multimorbidity and FHSQ outcomes at 3 and 6 months, which are unadjusted and adjusted for baseline FHSQ domain, age and BMI. (DOCX 14 kb)

Additional file 6: Graph of FHSQ domain scores for closed versus open cohorts. Graphical representation of median FHSQ domain scores at each study time point for closed versus open cohorts. (TIF 252 kb)

Additional file 7: Graph of FHSQ domain change scores for closed versus open cohorts. Graphical representation of median FHSQ domain change scores at each study follow-up for closed versus open cohorts. (TIF 132 kb)

## Data Availability

Datasets are available from the corresponding author upon reasonable request.

## Abbreviations

BMI: Body mass index
EQ5D-5 L: Euro quality of life (Euroqol) five dimension 5 level questionnaire
EQ-VAS: Euro quality of life (Euroqol) questionnaire visual analogue scale
FHSQ: Foot health status questionnaire
GG&C: Greater Glasgow and Clyde
GP: General practitioner
HRQoL: Health-related quality of life
IQR: Inter-quartile range
MID: Minimal important difference
MSK: Musculoskeletal
NHS: National Health Service
NRS: Numerical rating scale
PRCS: Patient rating of change scale
PROM: Patient reported outcome measure
PROMfoot: Patient Reported Outcome Measure Foot Study
REDCap: Research Electronic Data Capture
SCQ: Self-assessment comorbidity questionnaire
SD: Standard deviation
UK: United Kingdom
