# Supplementary material for: The impact of multimorbidity on foot health outcomes in podiatry patients with musculoskeletal foot pain: a prospective observational study
Source: J Foot Ankle Res. 2019 Jul 3;12:36. doi: 10.1186/s13047-019-0346-x (PMC6609344; doi:10.1186/s13047-019-0346-x)
Supplement: Supplementary file 1 — Table of demographic characteristics of the whole sample versus the closed cohort. Table comparing demographic characteristics between the whole sample which includes some missing data, and the closed cohort with complete data at each time point. (DOCX 12 kb) [file 13047_2019_346_MOESM1_ESM.docx]

|  | Whole sample | Closed cohort |
| --- | --- | --- |
| Number of participants, n (%) | 115 (100) | 64 (100) |
| N (%) female | 68 (59.1) | 38 (59.4) |
|  |  |  |
| Proportions of multimorbidity |  |  |
| N (%) no conditions | 26 (22.6) | 9 (14.1) |
| N (%) single condition | 28 (24.3) | 19 (29.7) |
| N (%) multiple conditions | 61 (53.0) | 36 (56.3) |
|  |  |  |
| Age in years, mean (SD) | 55.04 (11.56) | 56.44 (11.49) |
| BMI, mean (SD) | 35.41 (12.3) | 34.46 (12.79) |
|  |  |  |
| EQ-5D-5L Index, mean (SD) | 0.55 (0.31) | 0.58 (0.30) |
| EQ-5D-5L VAS, mean (SD) | 66.69 (25.74) | 68.9 (26.39) |
|  |  |  |
| SIMD lowest 2 quintiles, n (%) | 48 (41.7) | 20 (31.3) |
|  |  |  |
| Employment status |  |  |
| Paid or self-employed full time | 39 (33.9) | 21 (32.8) |
| Paid or self-employed part time | 17 (14.8) | 10 (15.6) |
| Voluntary work | 1 (0.9) | 0 |
| Looking for work | 2 (1.7) | 0 |
| Student in further or higher education | 2 (1.7) | 1 (1.6) |
| Looking after the home or family | 4 (3.5) | 3 (4.7) |
| Wholly retired | 30 (26.1) | 20 (31.3) |
| Permanently unable to work | 10 (8.7) | 5 (7.8) |
| Other | 5 (4.3) | 3 (4.7) |
| Prefer not to answer | 2 (1.7) | 1 (1.6) |
|  |  |  |
