# Supplementary material for: The impact of multimorbidity on foot health outcomes in podiatry patients with musculoskeletal foot pain: a prospective observational study
Source: J Foot Ankle Res. 2019 Jul 3;12:36. doi: 10.1186/s13047-019-0346-x (PMC6609344; doi:10.1186/s13047-019-0346-x)
Supplement: Supplementary file 2 — Table of FHSQ domain scores for the closed cohort. Table displays FHSQ domain scores at each study time point and results of between group sensitivity analysis. (DOCX 12 kb) [file 13047_2019_346_MOESM2_ESM.docx]

|  | FHSQpain, median (IQR) | FHSQfunction, median (IQR) | FHSQfootwear, median (IQR) | FHSQhealth, median (IQR) |
| --- | --- | --- | --- | --- |
| Baseline |  |  |  |  |
| No condition | 48.13 (40.0) | 62.50 (34.38)* | 16.67 (62.5) | 72.5 (67.5) |
| Single condition | 35.63 (41.25) | 68.75 (31.25)*‡ | 50.0 (41.67) | 42.5 (47.5) |
| >1 conditions | 29.38 (47.81) | 40.63 (56.25)* | 29.17 (41.67) | 25.0 (60.0) |
|  |  |  |  |  |
| 3 months |  |  |  |  |
| No condition | 60.63 (30.31)* | 87.5 (31.25)** | 50.0 (70.84) | 55.0 (73.75)* |
| Single condition | 41.25 (49.37)* | 87.5 (37.5)**‡‡ | 50.0 (58.33) | 60.0 (35.0)*‡ |
| >1 conditions | 35.32 (43.12)*† | 34.38 (60.94)**†† | 25.0 (47.92) | 25.0 (60.0)*† |
|  |  |  |  |  |
| 6 months |  |  |  |  |
| No condition | 65.63 (27.81) | 93.75 (25.0)** | 41.67 (66.66)* | 85.0 (46.25)* |
| Single condition | 41.25 (49.38) | 87.5 (25.0)**‡‡ | 41.67 (50.0)*‡‡ | 42.5 (60.0)* |
| >1 conditions | 35.63 (48.90) | 43.75 (62.50)**†† | 20.84 (41.67)* | 25.0 (60.0)*†† |
| Kruskall-Wallis tests significant at **p<0.01 and *p<0.05  Post-hoc Mann-Whitney tests significant at ‡‡p<0.01 and ‡p<0.05 for Single Condition versus >1 conditions group comparison  Post-hoc Mann-Whitney tests significant at ††p<0.01 and †p<0.05 for No conditions versus >1 conditions comparison | | | | |
