# Supplementary material for: The impact of multimorbidity on foot health outcomes in podiatry patients with musculoskeletal foot pain: a prospective observational study
Source: J Foot Ankle Res. 2019 Jul 3;12:36. doi: 10.1186/s13047-019-0346-x (PMC6609344; doi:10.1186/s13047-019-0346-x)
Supplement: Supplementary file 3 — Table of FHSQ domain change scores scores for the closed cohort. Table displays FHSQ domain change scores at each study follow-up and results of between group sensitivity analysis. (DOCX 12 kb) [file 13047_2019_346_MOESM3_ESM.docx]

|  | FHSQpain, median (IQR) | FHSQfunction, median (IQR) | FHSQfootwear, median (IQR) | FHSQhealth, median (IQR) |
| --- | --- | --- | --- | --- |
| 0 to 3 months |  |  |  |  |
| No condition | -6.26 (45.63) | 0.0 (21.88) | -16.67 (25.0)* | 0 (12.50) |
| Single condition | -5.63 (21.88) | -6.25 (31.25) | 0.0 (25.0)* | 0 (25.0) |
| >1 conditions | -5.01 (18.12) | 0.0 (12.5) | 0.0 (25.0)*† | 0 (21.88) |
|  |  |  |  |  |
| 0 to 6 months |  |  |  |  |
| No condition | -1.88 (46.26) | -18.75 (25.0) | -25.0 (45.48)* | 0.0 (27.5) |
| Single condition | -0.01 (41.26) | -18.75 (25.0) | 0.0 (25)* | 0.0 (50.0) |
| >1 conditions | -6.26 (21.72) | 0 (10.94) | 0.0 (16.67)*† | 0.0 (16.25) |
| Kruskall-Wallis tests significant at *p<0.05  Post-hoc Mann-Whitney tests significant at †p<0.05 for No conditions versus >1 conditions comparison | | | | |
