# Supplementary material for: The impact of multimorbidity on foot health outcomes in podiatry patients with musculoskeletal foot pain: a prospective observational study
Source: J Foot Ankle Res. 2019 Jul 3;12:36. doi: 10.1186/s13047-019-0346-x (PMC6609344; doi:10.1186/s13047-019-0346-x)
Supplement: Supplementary file 4 — Cross-tabulation contingency table of perceptions of foot pain change according to multimorbidity group. Table displays results of cross-tabulation frequencies and chi-square sensitivity analysis of associations between group membership and perceptions of foot pain change. (DOCX 12 kb) [file 13047_2019_346_MOESM4_ESM.docx]

|  |  | No change/ deteriorated | Improved | Cramer’s V | p-value |
| --- | --- | --- | --- | --- | --- |
| 0 to 3 months (n=64) |  |  |  |  |  |
| No condition | Yes | 5 (12.8) | 4 (16.0) | 0.20 | 0.263 |
| Single condition | Yes | 9 (23.1) | 10 (40.0) |  |  |
| Multimorbidity | Yes | 25 (64.1) | 11 (44.0) |  |  |
|  |  |  |  |  |  |
| 0 to 6 months (n=64) |  |  |  |  |  |
| No condition | Yes | 4 (10.3) | 5 (20.8) | 0.23 | 0.202 |
| Single condition | Yes | 10 (25.6) | 9 (37.5) |  |  |
| Multimorbidity | Yes | 25 (64.1) | 10 (41.7) |  |  |
