# Supplementary material for: The impact of multimorbidity on foot health outcomes in podiatry patients with musculoskeletal foot pain: a prospective observational study
Source: J Foot Ankle Res. 2019 Jul 3;12:36. doi: 10.1186/s13047-019-0346-x (PMC6609344; doi:10.1186/s13047-019-0346-x)
Supplement: Supplementary file 5 — Unadjusted and adjusted associations between multimorbidity and foot health outcomes. Table displays results of multivariate linear regression analyses for associations between multimorbidity and FHSQ outcomes at 3 and 6 months, which are unadjusted and adjusted for baseline FHSQ domain, age and BMI. (DOCX 14 kb) [file 13047_2019_346_MOESM5_ESM.docx]

| Variable | FHSQ pain 3 months | | | FHSQ pain 6 months | | |
| --- | --- | --- | --- | --- | --- | --- |
| Unadjusted | *B* | *SE B* | *β* | *B* | *SE B* | *β* |
| Multimorbidity | -17.95 | 7.529 | -0.32* | -25.96 | 7.90 | -0.48** |
| Single condition | -11.13 | 8.82 | -0.17 | -18.20 | 8.90 | -0.31* |
| Adjusted |  |  |  |  |  |  |
| Multimorbidity | -2.64 | 5.67 | -0.05 | -16.96 | 6.90 | -0.31* |
| Single condition | -3.37 | 6.28 | -0.05 | -13.91 | 7.30 | -0.24 |
| Baseline FHSQ pain | 0.85 | 0.09 | 0.77** | 0.63 | 0.63 | 0.60** |
| Age | -0.35 | 0.20 | -0.14 | -0.16 | -0.16 | -0.07 |
| BMI | 0.13 | 0.18 | 0.06 | -0.03 | -0.03 | -0.02 |
| FHSQ Pain 3 months*, R*^2^ = 0.06 for unadjusted, *R*^2^ = 0.56 for adjusted; FHSQ Pain 6 months, *R*^2^ = 0.12 for unadjusted, *R*^2^ = 0.46 for adjusted. | | | | | | |
|  |  |  |  |  |  |  |
|  | FHSQ function 3 months | | | FHSQ function 6 months | | |
| Unadjusted | *B* | *SE B* | *β* | *B* | *SE B* | *β* |
| Multimorbidity | -33.63 | 8.11 | -0.51** | -35.36 | 8.67 | -0.55** |
| Single condition | -5.36 | 9.50 | -0.07 | -5.07 | 9.68 | -0.07 |
| Adjusted |  |  |  |  |  |  |
| Multimorbidity | -17.09 | 6.51 | -0.26* | -21.92 | 6.15 | -0.35** |
| Single condition | -7.89 | 6.84 | -0.10 | -9.90 | 6.50 | -0.14 |
| Baseline FHSQ function | -0.74 | 0.08 | 0.71** | 0.72 | 0.07 | 0.71** |
| Age | 0.19 | 0.21 | 0.06 | -0.12 | 0.18 | -0.05 |
| BMI | 0.05 | 0.19 | 0.02 | -0.04 | 0.17 | -0.02 |
| FHSQ Function 3 months*, R*^2^ = 0.21 for unadjusted, *R*^2^ = 0.65 for adjusted; FHSQ Function 6 months, *R*^2^ = 0.25 for unadjusted, *R*^2^ = 0.69 for adjusted. | | | | | | |
|  | | | | | | |
| Variable | FHSQ footwear 3 months | | | FHSQ footwear 6 months | | |
| Unadjusted | *B* | *SE B* | *β* | *B* | *SE B* | *β* |
| Multimorbidity | -9.56 | 7.70 | -0.16 | -16.87 | 9.23 | -0.27 |
| Single condition | 3.94 | 8.94 | 0.06 | 4.27 | 10.22 | 0.06 |
| Adjusted |  |  |  |  |  |  |
| Multimorbidity | -6.30 | 6.70 | -0.11 | -15.31 | 7.66 | -0.45* |
| Single condition | 0.51 | 7.60 | 0.01 | -1.98 | 8.27 | -0.03 |
| Baseline FHSQ footwear | 0.60 | 0.10 | 0.56** | 0.63 | 0.10 | 0.57** |
| Age | 0.34 | 0.24 | 0.13 | 0.03 | 0.24 | 0.01 |
| BMI | 0.23 | 0.21 | 0.10 | 0.03 | 0.22 | 0.01 |
| FHSQ Footwear 3 months*, R*^2^ = 0.04 for unadjusted, *R*^2^ = 0.35 for adjusted; FHSQ Footwear 6 months, *R*^2^ = 0.10 for unadjusted, *R*^2^ = 0.43 for adjusted. | | | | | | |
|  |  |  |  |  |  |  |
|  | FHSQ general 3 months | | | FHSQ general 6 months | | |
| Unadjusted | *B* | *SE B* | *β* | *B* | *SE B* | *β* |
| Multimorbidity | -16.60 | 8.03 | -0.27* | -24.30 | 9.74 | -0.37* |
| Single condition | -1.13 | 9.50 | -0.02 | -10.01 | 10.79 | -0.14 |
| Adjusted |  |  |  |  |  |  |
| Multimorbidity | 0.32 | 5.71 | 0.01 | -14.03 | 7.38 | -0.21 |
| Single condition | 2.17 | 6.37 | 0.03 | -10.31 | 7.90 | -0.14 |
| Baseline FHSQ general | 0.76 | 0.07 | 0.77** | 0.74 | 0.09 | 0.70** |
| Age | -0.20 | 0.20 | -0.07 | -0.01 | 0.23 | -0.00 |
| BMI | 0.21 | 0.20 | 0.09 | 0.30 | 0.21 | 0.12 |
| FHSQ General 3 months*, R*^2^ = 0.07 for unadjusted, *R*^2^ = 0.60 for adjusted; FHSQ General 6 months, *R*^2^ = 0.09 for unadjusted, *R*^2^ = 0.53 for adjusted. | | | | | | |
