# Supplementary figures and images for: The impact of multimorbidity on foot health outcomes in podiatry patients with musculoskeletal foot pain: a prospective observational study
Source: J Foot Ankle Res. 2019 Jul 3;12:36. doi: 10.1186/s13047-019-0346-x (PMC6609344; doi:10.1186/s13047-019-0346-x)

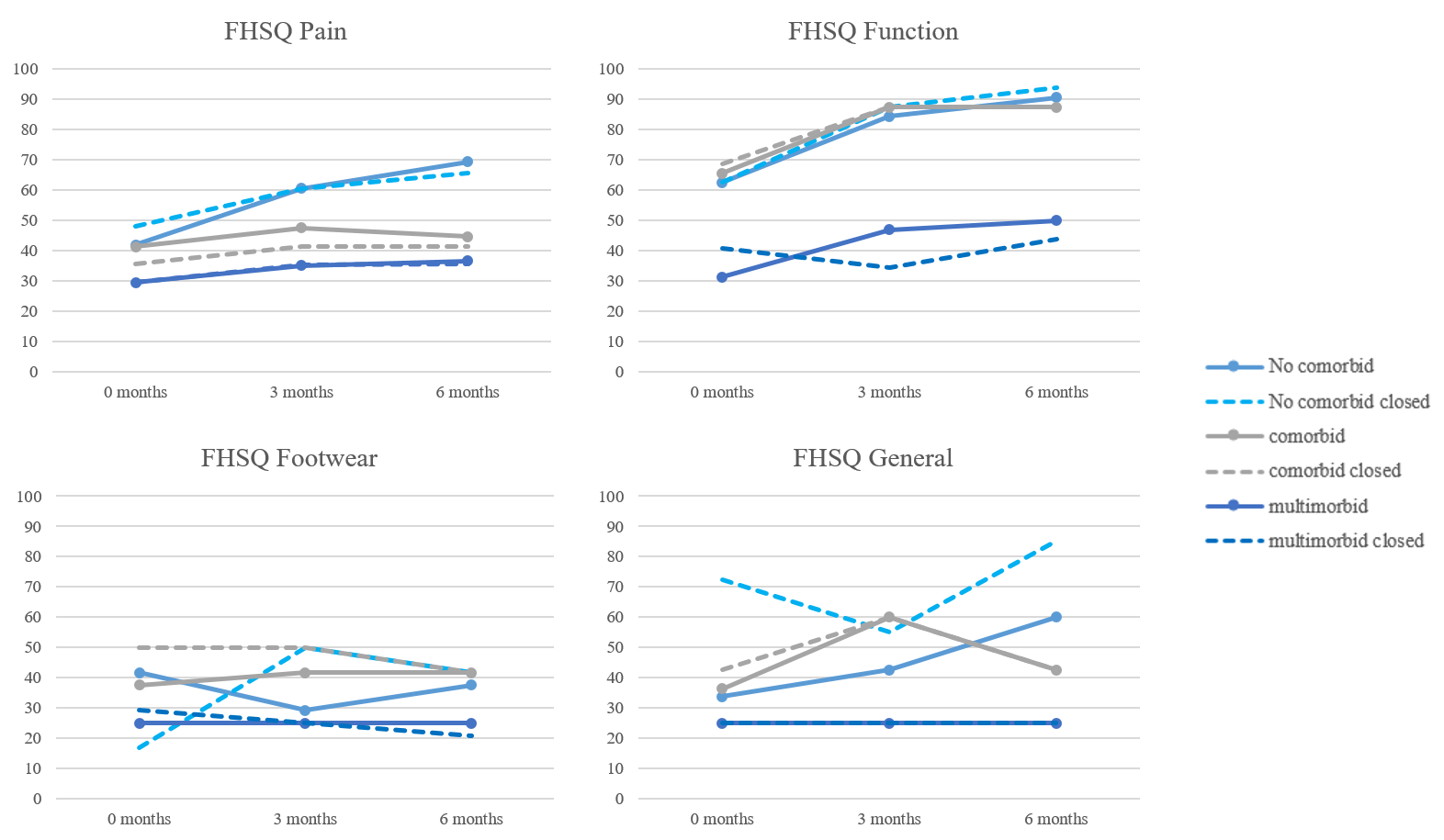

Supplement: Supplementary file 6 — Graph of FHSQ domain scores for closed versus open cohorts. Graphical representation of median FHSQ domain scores at each study time point for closed versus open cohorts. (TIF 252 kb) [file 13047_2019_346_MOESM6_ESM.tif]

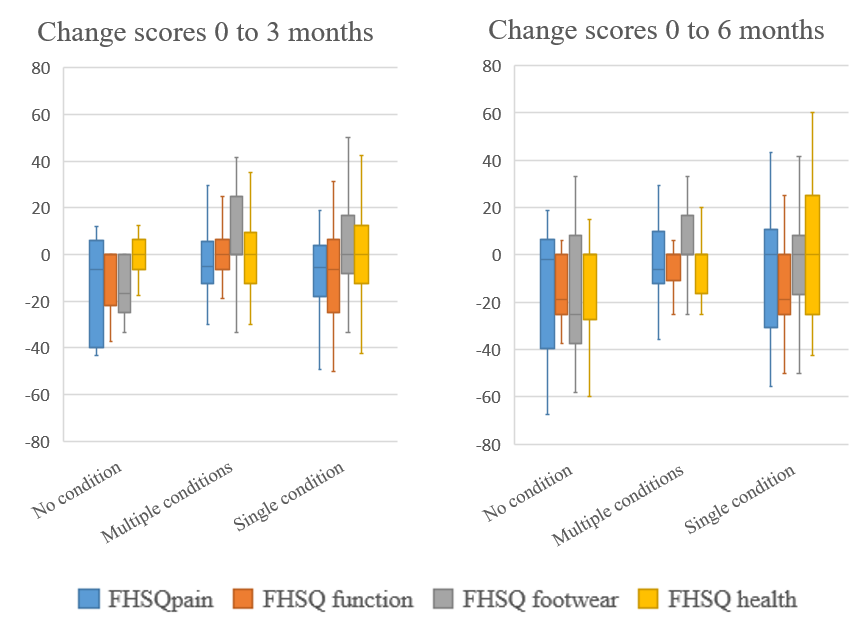

Supplement: Supplementary file 7 — Graph of FHSQ domain change scores for closed versus open cohorts. Graphical representation of median FHSQ domain change scores at each study follow-up for closed versus open cohorts. (TIF 132 kb) [file 13047_2019_346_MOESM7_ESM.tif]
